# Correspondence: Reply to ‘The experimental requirements for a photon thermal diode’

**DOI:** 10.1038/ncomms16136

**Published:** 2017-08-21

**Authors:** Zhen Chen, Carlaton Wong, Sean Lubner, Shannon Yee, John Miller, Wanyoung Jang, Corey Hardin, Anthony Fong, Javier E. Garay, Chris Dames

**Affiliations:** 1 Department of Mechanical Engineering, University of California, Berkeley, California 94720, USA; 2 Department of Mechanical Engineering, University of California, Riverside, California 92521, USA

*Nature Communications* 8:16136 doi: 10.1038/ncomms16136 (2017); Published: 21 August 2017

Budaev^1^ correctly identifies a fundamental symmetry error in several crucial experiments in our recent study^2^, specifically the results presented in Fig. 3c (the three filled and four striped bars, labeled ‘Col. 1’ and ‘Col. 2’, respectively) and Fig. 4. A suitable configuration for those measurements should have included identical (mirror-imaged) collimators at both hot and cold sides, as in Fig. 1a here. However, the actual experiments omitted the cold-side collimator, a choice made for experimental simplicity and which we believed was acceptable at the time based on a simple thermal model^3^ and the fact that *T*_BBC_^4^≫*T*_∞_^4^, where *T*_BBC_ and *T*_∞_ are the temperatures of the blackbody (BB) cavity and cold-side plate, respectively. However, upon careful reconsideration we now find that thermal model to be flawed, and we believe that omitting the cold-side collimator invalidated several key measurements. We provide a more detailed discussion of that thermal estimate, and the reasons for its failure, elsewhere^3^.

Although we believe the heat flow (*Q*) measurements in ref. 2 were accurate for all of the configurations presented, due to the symmetry error none of those experimental configurations were actually relevant to the following two major claims, which therefore are invalidated for lack of experimental support: First, that these experiments demonstrated a thermal diode. Second, that the ‘inelastic thermal collimation’ mechanism is a suitable nonlinearity for realizing thermal rectification when combined with asymmetric scattering structures (for example, copper pyramids or etched triangular pores in silicon).

The symmetry error^1^ does not apply to the experiments without thermal collimation, specifically the results presented in Fig. 3c for photons (the six leftmost, unfilled bars) and Supplementary Fig. 12 for phonons. Therefore, the last major conclusion of ref. 2 remains well-supported by the original experiments: Asymmetric scattering alone is insufficient to achieve thermal rectification.

The rest of this Reply is devoted to another problem which we have only recently realized: a fundamental issue with the inelastic thermal collimation concept based on absorption, thermalization, and re-emission, as exemplified by the perforated graphite plate approach used in ref. 2. This leads us to now conclude that if it had used a correct two-plate symmetry as depicted here in Fig. 1a, the thermalizing graphite plate scheme as originally conceived^2^ could not rectify.

The essence of the graphite plate approach is radiation absorption and re-emission by a solid plate of infinite thermal conductivity, as exemplified by Supplementary Fig. 4 of ref. 2. The motivating insight is that when analyzed as part of its adjacent BB reservoir, the combined effect of (BB+collimator) is to convert a local boundary condition into a nonlocal one (or linear into nonlinear in the language originally used in ref. 2). This is depicted here in Fig. 1b, corresponding closely to Supplementary Fig. 5c of ref. 2. This shows how the graphite plate next to BB_1_ can convert the local equilibrium Bose-Einstein statistics *f*_BE_(*T*_1_) to a nonlocal, non-equilibrium reservoir boundary condition *f*_NE,1_(*T*_1_,*T*_2_), at the boundary between (BB_1_+plate_1_) and the test section Σ_A_, as shown here in Fig. 1b. As noted below Supplementary Equation 5 of ref. 2, this functional form *f*_NE,1_(*T*_1_,*T*_2_) is a necessary condition for the heat transfer response function *Q*(*T*_1_,*T*_2_) through the test section Σ_A_ to be non-symmetric upon the exchange *T*_1_↔*T*_2_. Analogous statements hold for the other boundary condition, at the interface between Σ_A_ and (BB_2_+plate_2_).

However, the heat transfer analysis could just as well combine the graphite plates with the pyramids as a larger alternate test section, not considered in ref. 2 but indicated here in Fig. 1c as Σ_B_. Because the only distinction between Fig. 1b, c is in how the control volumes are drawn, with no changes to the physical system, clearly both approaches must give the same heat transfer response function *Q*(*T*_1_,*T*_2_). Yet because the boundary conditions in Fig. 1c are now ideal blackbodies, Σ_B_ can be analyzed rigorously using radiation network analysis^4^, as indicated schematically here in Fig. 2. This network analysis accounts for the direct and indirect radiative exchanges between numerous differential areas which cover all surfaces, including pyramids and graphite plates. The approach can be generalized to handle surfaces with any combination of diffuse (for example, the BBs and graphite plates) and specular (for example, the copper pyramids and sidewalls) character^4^.

The essential point is that the resulting matrix formulation of the heat transfer problem^4^ is fundamentally a linear relationship in terms of the BB emissive powers *E*_b,i_=*σT*_i_^4^, where σ is the Stefan-Boltzmann constant. Specifically, the heat transfer can be expressed as *Q*(*T*_1_,*T*_2_)=[*E*_b_(*T*_1_)−*E*_b_(*T*_2_)]/*R*_eff_, where the effective radiation resistance *R*_eff_ is independent of *T*_1_ and *T*_2_. Therefore, the radiation network analysis of Σ_B_ shows that the heat transfer must be anti-symmetric upon the exchange *T*_1_↔*T*_2_, and this system cannot rectify.

Thus, even though from the Σ_A_ analysis the graphite plate approach gives the reservoir boundary conditions the nonlocal functional form *f*_NE,1_(*T*_1_,*T*_2_) that is necessary to enable rectification, the parallel analysis using Σ_B_ reveals that this particular approach is still not sufficient, and cannot rectify. We conclude that the *f*_NE,1_ and *f*_NE,2_ obtained by the thermalizing graphite plate approach exhibit a special symmetry which renders them insufficient for rectification, a possibility briefly identified just below in Supplementary Equation 5 of ref. 2 but not considered further there.

Last, we briefly comment on the implications of this type of radiation resistor network analysis for the experimental configurations presented in ref. 2. First, for the experiments without a collimator (the six leftmost, unfilled bars of Fig. 3c for photons, and Supplementary Fig. 12 for phonons), this network analysis confirms what was previously demonstrated by Supplementary Equations 1–4, namely, that such a system cannot rectify upon the exchange *T*_1_↔*T*_2_. Thus, this finding remains well-supported both theoretically and experimentally by the original work^2^.

On the other hand, the network analysis does not bear directly on the experiments with a single collimator (the three filled and four striped bars of Figs 3c and 4) because of the way they were misconfigured. What was required was to measure a single device, call it Σ_C_, in two different thermal bias directions. Because the key experiments in ref. 2 used only a single collimator, this Σ_C_ can be understood as the copper pyramids plus one graphite plate located near the pyramids’ points. In this case the linear network argument just presented shows that one expects |*Q*_ΣC_(*T*_1_,*T*_2_)|=|*Q*_ΣC_(*T*_2_,*T*_1_)|, that is, no rectification. But what was actually measured were two different devices: in Σ_C_, the graphite plate was located near the pyramids’ points, while in Σ_D_, the plate was located near the pyramids’ bases. For example, for the experiments using the larger-holed collimator designated ‘Col. 1’ in Fig. 3c, the red and blue bars correspond to Σ_C_ and Σ_D_, respectively. Thus, these experiments compared *Q*_ΣC_(*T*_1_,*T*_2_) to *Q*_ΣD_(*T*_2_,*T*_1_), rather than *Q*_ΣC_(*T*_1_,*T*_2_) to *Q*_ΣC_(*T*_2_,*T*_1_). Although they do not represent a diode, the measurements showing |*Q*_ΣC_(*T*_1_,*T*_2_)|>|*Q*_ΣD_(*T*_2_,*T*_1_)| in Fig. 3c remain valid and are consistent with the original ideas about how the perforated graphite plate skews the radiation towards more forward-peaked directions (for example, Supplementary Figs 4 and 8 and Fig. 1b of ref. 2), which then transmits through the pyramids more easily when this radiation is incident towards the pyramids’ points (for example, Supplementary Figs 2 and 3 and Fig. 1a of ref. 2). Yet as pointed out by Budaev^1^, due to the internal reconfiguration which broke the symmetry required of a passive device, this set of measurements does not represent a thermal diode.

## Additional information

**How to cite this article:** Chen, Z. *et al*. Correspondence: Reply to ‘The experimental requirements for a photon thermal diode’. *Nat. Commun.* **8**, 16136 doi: 10.1038/ncomms16136 (2017).

**Publisher’s note:** Springer Nature remains neutral with regard to jurisdictional claims in published maps and institutional affiliations.

## Figures and Tables

**Figure 1:**
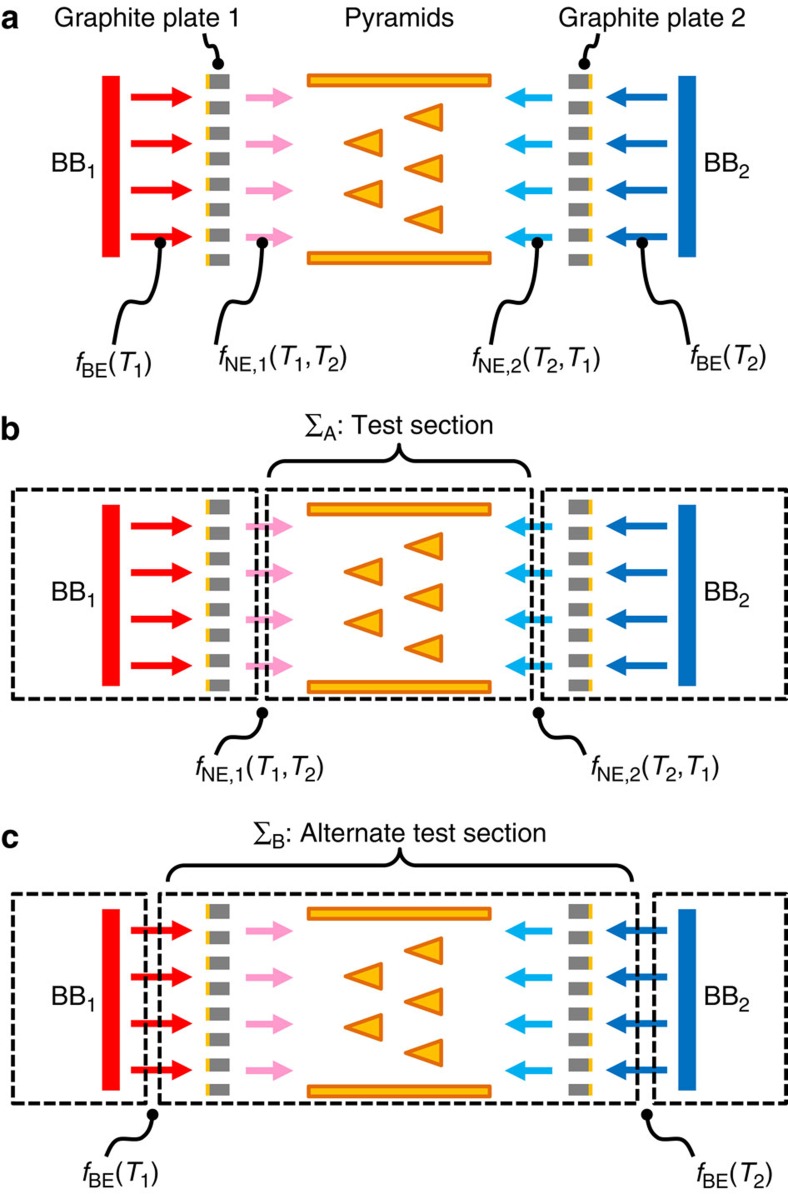
Alternative analyses reveal a fundamental problem with the thermalizing graphite plate concept for inelastic thermal collimation. (**a**) Schematic of a configuration with proper symmetries^1^, using two mirror-imaged thermal collimators. The heat transfer response function *Q*(*T*_1_,*T*_2_) can be analyzed using either of the control volumes depicted in **b**,**c**. (**b**) In the original work (for example, Supplementary Fig. 5c of ref. 2), each graphite plate was considered part of its adjacent BB, corresponding to non-equilibrium reservoir boundary conditions *f*_NE,1_(*T*_1_,*T*_2_) and *f*_NE,2_(*T*_2_,*T*_1_). (**c**) Alternatively, the graphite plates can be considered with the pyramids as Σ_B_. In this case, the reservoir boundary conditions are perfect blackbodies with equilibrium Bose-Einstein statistics *f*_BE_(*T*_1_) and *f*_BE_(*T*_2_), analyzed further here in Fig. 2. Clearly the *Q*(*T*_1_,*T*_2_) function must be the same whether calculated using **b** or **c**.

**Figure 2:**
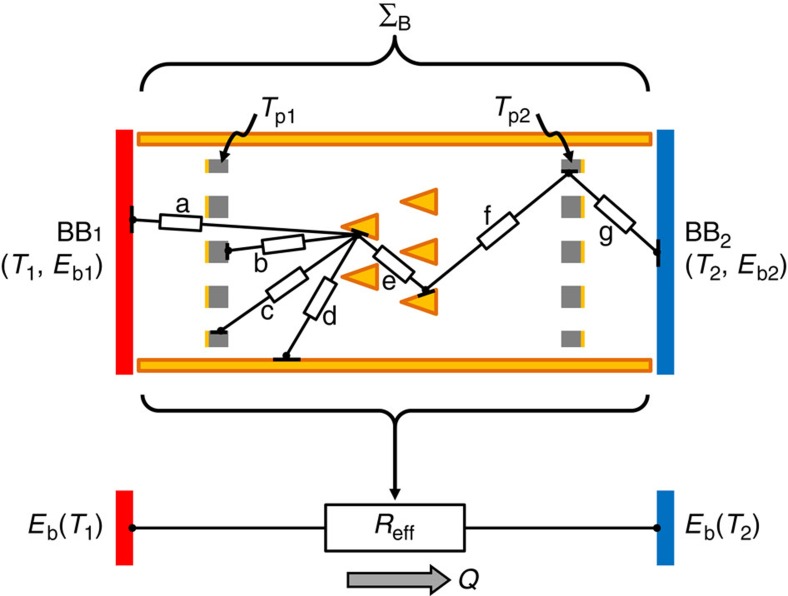
Implications of a radiation resistor network analysis of Σ_B_ from Fig. 1c. *Top*: All surfaces are discretized into differential areas and joined by numerous radiation resistors, a very few of which are indicated schematically as a–g. Each graphite plate has infinite thermal conductivity and thermalizes to its own unknown temperature (*T*_p1_, *T*_p2_), while the pyramids and test section sidewalls are taken as perfectly reflecting (diffuse and/or specular), and the two reservoirs as ideal BBs. *Bottom*: For these conditions, it is well known that the heat transfer analysis can be expressed as a linear system of equations^4^, corresponding to a single effective resistor *R*_*eff*_ between the driving potentials *E*_b1_ and *E*_b2_. Thus, the response function *Q*(*T*_1_,*T*_2_) must be anti-symmetric upon the exchange *T*_1_↔*T*_2_. We conclude that if it had used the correct two-plate symmetry as depicted here in Fig. 1a, the thermalizing graphite plate scheme as originally conceived^2^ could not rectify.
